# The Hospitals on Christmas Day

**Published:** 1898-01-01

**Authors:** 


### THE HOSPITALS ON CHRISTMAS DAY.

Year by year, as Christmas comes round, and we pay our annual visit to the hospitals of London, we become more and more impressed with the humanising and Christian influence of these great institutions. Years ago a west-end vicar startled the world by instituting services on Christmas Day for the solitary. We venture to think that any person so unfortunate as to find himself alone in London on Christmas Day could not do better than rise early and spend a day with the patients and nurses, the students and the doctors in one or more of the metropolitan hospitals. Here at all times, and especially during the festival of Christmas, love presides, and the atmosphere of tenderness and unselfishness so makes itself felt that the heart of Scrooge himself would be warmed and revivified by such a visit. This year the largest hospital in London has taken a new departure, owing to the dislike which the present chairman is known to have to Christmas decorations in hospital wards. Instead of the elaborate devices which have heretofore prevailed only sufficient holly has been put about the wards to show that it has not been banished altogether. The chairman's objection to decorations seems to be based upon two grounds. First, that the average day's work of a nurse is eleven hours, and she can therefore spare no further time for decorations; secondly, that the decorations are not desirable from a sanitary point of view. The first objection applies mainly, if not entirely, to the London Hospital, owing to the large number of beds and the comparatively small number of students. In other great hospitals there are a much greater proportion of medical students, and the *esprit* amongst these gentlemen is so excellent that the Christmas decorations and the amusement of the patients are undertaken by them with a whole-hearted devotion and energy beyond all praise. As to the sanitary objection, it can have little weight with practical men in charge of a well-regulated hospital, where arrangements are made which provide that the decorations shall only remain up for a strictly limited period, and that all due precautions shall be taken to remove any objection of this nature.

There is, however, in some hospitals a great evil connected with the Christmas decorations of the wards which ought to be removed without delay. We allude to a few hospitals where, every Christmas, the decoration of the wards has to be done mainly if not entirely at the cost of the sisters and the nurses. When it is understood that the cost of lighting up one single ward in some hospitals averages from 15s. to 20s. a night, it can readily be understood that the decorations may entail an expenditure upon each member of the nursing staff which cannot be much less in amount than one month's salary. It is certainly necessary for every chairman of every hospital to go into this question of the expenditure of the sisters and nurses on matters of this kind, and also in regard to clothing supplied to necessitous patients, because at the present time, and still more so in past years, the burden upon them of providing such things has been so great as to render it impossible for some sisters and nurses to save anything out of their earnings as a protection for themselves against the day of sickness or old age. We welcome the evidence which is increasing everywhere that the governors recognise that every hospital should have at its head a chairman of energy and intelligence who will interest himself in these matters, and, by studying the practice at other hospitals, gradually introduce changes into the institution for the administration of which he is responsible which will put a stop to practices and evils of the kind we have referred to.

### Middlesex Hospital.

Every visitor to the Middlesex Hospital on the 25th ult. became aware that there was going to be a great Christmas tree there on Christmas Day from the fact that the board-room door is immediately opposite the entrance, and the first thing noticed was the Christmas tree in process of being dressed for the festival. We have always felt in inspecting hospitals that a visit to the secretary's office or the matron's room very often revealed at a glance the condition of the management. If these officers are on duty, if the surroundings of their daily work are orderly and methodical, then you may be certain that the whole institution is well managed. Where either or both of these officers consider themselves first, where they are too often absent on festival occasions, where either of them is away from the hospital on Christmas Day or other great festivals, there the average of efficiency is sure to be low. Mr. Melhado was busily engaged in his office, and Miss Thorold was absorbed in the work of superintending the dressing of the Christmas tree. At great expense the whole internal arrangements of this hospital have been renovated and renewed in recent years, and there are no wards in London which present a better appearance, which are better lighted, or where the fittings and nursing arrangements generally are more efficient than at Middlesex. Another test is the appearance of the patient. In some hospitals, we regret to say, one hospital being unfortunately notorious for this failure, the nurses regard the patients more as cases than as human beings, and there the aspect of the patient is almost necessarily one of discomfort and neglect. At the Middlesex Hospital every patient was happy and contented, lovingly cared for, and more comfortable than many of them could possibly have been anywhere else in the world. Of course, we visited the children's ward, which was very tastefully decorated, and the sister of which has an artistic eye which should entitle her to a visit from some of the artists to the illustrated papers, who would find more than one design in decoration well worthy of reproduction in their columns. In another ward we came across an old type of sister—one of those good, thoughtful, sympathetic, efficient souls, who makes every patient in her ward bless her, and who never wants to leave it until the last moment. Much may be said, no doubt, in favour of the modern nurse, but for real genuine kindness and efficiency there is probably nothing so completely satisfactory as the best specimen of the old type, of which this Middlesex sister is one. We found two nurses in one of the wards, victims of typhoid fever; one of them was a staff-nurse, who was very ill, the other a member of the Nurses' Co-operation. The latter was high in her praises of the care and attention she had received at Middlesex Hospital, and her appearance certainly did credit to her treatment, as she was in the first week of the convalescent stage, and rejoiced to be permitted to have a mouthful of turkey on Christmas Day in honour of the festival. It is only right to congratulate the authorities of the Middlesex Hospital upon the splendid appearance and perfect order of the whole hospital, although many of the patients had their friends to visit them in the wards. As a gentleman who was with us remarked, the appearance presented could not have been better had a royal personage been expected to visit the hospital. Indeed, Middlesex Hospital on Christmas Day, 1897, looked as if "it was being run for a parade." This is remarkable evidence of the efficiency of the administration, seeing that there were only eight vacant beds in the whole hospital, and that the work in consequence must have been exceptionally heavy.

### The London Hospital.

We made our way at once to Queen and Beatrice Wards, where there are fifty children at least. On entering we at once realised that the clever and able drawing by Lucien Davis, R.I., in this week's *Illustrated London News* of this ward does not represent its ordinary arrangements. In the picture the cots are shown back to back, and there is very little space between the double row of cots in the middle of the ward and those on either side. In anticipation of any criticism on hygienic grounds it may be well to mention that the ward as pictured by Mr. Lucien Davis shows the beds arranged on the afternoon of the Christmas tree, when all the patients are wheeled into one ward, so that they may have an equal opportunity of seeing the tree and enjoying the entertainment provided for them. We were rather late in our visit; most of the children were tired out with the entertainments of the day, but some of them were still sprightly, running races or dancing to the music of a barrel organ which was being vigorously played at one end of the ward. We could wish that the well-to-do mothers of England would visit this great children's ward at the London Hospital on an ordinary working day, and then try to realise what the care and nursing of these little ones must mean to the devoted women who have charge of them, and to whose energy the marked efficiency of this ward is due. If the decorations at the London Hospital were comparatively scanty, the amusement provided for the patients in the wards was certainly not open to the same objection. The students and their friends had organised a series of entertainments between half-past four and half-past eight p.m., at which latter hour supper was provided for the performers in the residents' dining-room. The entertainers comprised the Boracic Brothers' waxworks, the Oxford House Glee Party, Mr. Barnard, the Cressal Band, Funny Phones, the Variety Trio, a dramatic performance entitled "Turn Him Out," the phonograph, and the Misses Thorpe. In addition, the patients in the various wards who were convalescent entertained those who were less fortunate by songs and recitations, some of which were excellently rendered. In the Mellish ward the sisters and nurses had provided a sumptuous entertainment for all visitors, and the scene presented was both attractive and animated. Certainly the uniforms, and especially the caps and aprons of the nurses of the London Hospital are much more artistic and attractive than those at Guy's, for instance, where an improvement in this direction might in mercy be introduced. We make this suggestion because the very name of Guy's Hospital offers an opportunity to the satirist under existing circumstances, who is unexpectedly brought face to face with a certain type of uniform where the dress is so plain that the beholder is involuntarily compelled to pity the wearer. We were fortunate enough to come across one of the assistant matrons of the London Hospital during our visit, without whose assistance we should have found it very difficult to see as much of the hospital, as we were with her kind assistance able to do, in the time at our disposal. One remarkable fact struck us, and that was the comparative smallness of the number of patients attending the casualty department not only on Christmas Day, but on the two days which immediately preceded it. This fact is one worthy of the serious consideration of the authorities, in conjunction with the comparatively slight ailments from which many of these casualty patients suffer. There can be little doubt that without any injury to the patients, but to their positive advantage as well as to that of the hospital, the number of casualty cases now admitted to treatment might be reduced, by a slight alteration in system, by at least one-half to two-thirds of the present numbers.

### Guy's Hospital.

On entering Guy's Hospital a novel sight presented itself to the delighted spectators who were fortunate enough to be there immediately after the wards had been lit up. Standing in the colonnade, and looking into the quads to left and right of it, a fairy scene was presented, the beauty of which is almost indescribable. Every ward displayed a variety of colour, sparkling with lights of varying hues and shades. Above the buildings was a heavy belt of thick fog, and we never remember to have come across a scene which presented the rejoicings which are typical of Christmas in so varied, so attractive, and so striking a form as that which rejoiced our eyes on entering the colonnade of Guy's Hospital on Christmas Day, 1897. The scene would have made a splendid etching, and it is one which a clever artist in pastels could make much of. The beauties of the two quads at Guy's Hospital on Christmas Day will long remain with us as a pleasant memory, for they showed at a glance the extraordinary energy which Guy's ever displays on all occasions when there is an opportunity to exhibit an efficient and capable staff, united as one man or one woman in doing good to others with all their hearts. Last year we gave so full an account of the beauties of the decorations of Guy's Hospital that it is unnecessary on the present occasion to repeat what we then said. Certainly no place in London ever keeps Christmas better than Guy's Hospital, or for that matter than most of the Metropolitan hospitals. Stephen ward was beautiful as a whole, and when taken in detail, thanks largely to the ingenuity and skill of a German patient, some of the decorations were wonders of design and beauty. In Martha, too, very great zeal had been displayed by the students, who had erected no less than three arches in the ward, each one of which differed from the other. In another ward, the name of which we do not remember, the entrance was guarded by one of the lightest and most effective ivy screens which we have ever seen. Indeed, it is not too much to say that for beauty of design and effect in execution, the Christmas decorations of Guy's Hospital were probably unsurpassed by any other decorations in connection with the celebration of Christmas in the Diamond Jubilee year.

Finally, whereas we did our best to foreshadow in the Special Christmas Number what was being attempted by the authorities of the various hospitals, we hope it may be understood that we have simply confined our description to the three hospitals it was possible to visit on Christmas Day, and that we hope next year to visit other hospitals, so as gradually to accomplish the pleasant task of making a thorough inspection of every London hospital at least once in our lives during the Christmas festival. From our point of view, and we believe from the point of view of every thinking man and woman of intelligence, nothing is more grateful to the feelings or more in character with the festival of Christmas than the spirit which dominates everybody in the great hospitals of this country, as anyone may judge for themselves who will take the trouble to enter almost any hospital on December 25th.

